# Yinchenhao Decoction Protects Against Intrahepatic Cholestasis During Pregnancy Through the miR-370-3p/TM9SF4/KIT Axis

**DOI:** 10.1155/bmri/3000226

**Published:** 2025-07-26

**Authors:** Hongxiu Jiang, Wenjing Yu, Xingran Tao, Qiao Yan, Guanlun Zhou, Chao Chen, Guorong Han

**Affiliations:** Department of Obstetrics and Gynecology, The Second Hospital of Nanjing, Affiliated to Nanjing University of Chinese Medicine, Nanjing, China

**Keywords:** intrahepatic cholestasis of pregnancy, KIT, miR-370-3p, TM9SF4, Yinchenhao decoction

## Abstract

**Objective:** The objective is to explore the potential pathogenesis and therapeutic mechanism of Yinchenhao decoction (YCHD) in intrahepatic cholestasis of pregnancy (ICP) by focusing on the regulatory role of exosomal miR-370-3p on target genes TM9SF4 and KIT.

**Methods:** Exosomes were isolated from the serum samples of normal pregnant women (control), patients with ICP, HTR-8/SVneo cells, and Sprague–Dawley (SD) pregnant rats via differential centrifugation. Characterization of these exosomes was performed using electron microscopy, nanoparticle tracking analysis (NTA), and western blotting. Quantitative reverse transcription PCR (qRT-PCR) and the bioinformatics tool starBase were used to identify miR-370-3p as a candidate miRNA. Dual-luciferase reporter assays were used to confirm that TM9SF4 and KIT are direct targets of miR-370-3p. An in vitro ICP cell model was established using HTR-8/SVneo cells to investigate the interactions between miR-370-3p and its targets. An animal model was established to validate the targeted regulation of miR-370-3p on TM9SF4 and KIT, as well as the therapeutic effect of YCHD *in vivo*.

**Results:** The exosomal miR-370-3p expression was significantly upregulated, whereas the TM9SF4 and KIT expressions were downregulated as demonstrated by qRT-PCR and western blot analyses. RNA pull-down assays confirmed a direct negative regulatory relationship between miR-370-3p and both TM9SF4 and KIT at the molecular level. Finally, the therapeutic potential of YCHD was verified by its ability to reverse the altered expression patterns of miR-370-3p, TM9SF4, and KIT in the animal ICP model.

**Conclusion:** Our study demonstrates that YCHD protects against ICP through the miR-370-3p/TM9SF4/KIT axis, suggesting miR-370-3p as a potential therapeutic target for ICP.

## 1. Introduction

Intrahepatic cholestasis of pregnancy (ICP) is the most common pregnancy-related liver disease. The primary clinical characteristic of ICP is pruritus and elevated total bile acid (TBA) in the second or third trimester of pregnancy, which usually disappears soon after delivery [1]. ICP is associated with increased perinatal complications such as premature delivery, respiratory distress, low Apgar scores, and stillbirth [2]. Determining serum TBA is the primary experimental evidence for diagnosing ICP, and it is also an important indicator for monitoring the condition and treatment effect [3]. However, its sensitivities and specificities are lacking. Thus, in recent years, the search for additional reliable predictors has gained popularity as a study topic and direction. The pathogenesis of ICP is complex and involves genetic, hormonal, immunological, and environmental factors [4]. Several studies have demonstrated that ICP is associated with placental immune dysregulation [5]. In addition, previous studies have implicated bile acid transport, hormone regulation, and VCAM-1 in the pathogenesis and development of ICP [6]. However, the precise mechanism of ICP remains to be clarified, and there are limited indicators available for ICP screening.

MicroRNAs (miRNAs) are highly conserved noncoding RNA molecules that play essential roles in various cellular processes, including organ formation, cell proliferation, and apoptosis [7, 8]. Exosomes are extracellular vesicles secreted by cells that carry miRNA. Previous studies have verified that circulating exosomes are the primary mechanism of miRNA transport [9]. Various miRNAs linked to ICP have been identified. Rao et al. reported that miR-148a mediates estrogen-induced cholestasis in ICP [10]. Feng et al. suggested that circ_0060731 induces the apoptosis of placental trophoblasts through the miR-21-5p-PDCD4/ESR1 pathway in ICP [11].

miR-370-3p has been identified as a novel biomarker for many diseases, including breast cancer [12], acute kidney injury [13], and osteosarcoma [14]. Peng et al. also demonstrated the diagnostic and therapeutic potential of miR-370-3p in hepatocellular carcinoma (HCC) metastasis [15]. Notably, the core pathological characteristics of ICP include abnormal apoptosis of placental trophoblasts and disturbance of bile acid metabolism, while miR-370-3p has been confirmed to participate in cell apoptosis by regulating target genes such as PDCD4 and ESR1, and affect disease progress through lipid metabolism–related pathways. These functions are highly consistent with the potential mechanisms of ICP, suggesting that miR-370-3p may play a cross-disease conservative regulatory role in ICP. Therefore, we selected miR-370-3p as the entry point for this study. A study unequivocally showed that the mTOR pathway is upregulated in the placenta with ICP. Bile acid can activate mTOR signaling, resulting in ER stress and decreased trophocyte viability [16]. Previous reports have suggested that TM9SF4 promotes the inactivation of mTOR, increasing autophagy flux and thus protecting cells from apoptosis and death [17]. Bosken et al. reported that c-kit/CD117 expression levels increased and that mTOR phosphorylation levels decreased in NK cells from patients with severe trauma [18]. These findings led us to hypothesize that TM9SF4 and KIT may be involved in regulating the physiological processes of ICP via mTOR signal transduction. Therefore, we selected TM9SF4 and KIT as target genes of miR-370-3p. HTR-8/SVneo human trophoblast cells were obtained by transfecting cells from human prepregnancy placental trophoblast explants and have been extensively employed in the investigation of trophoblast biology and placental functionality [19]. In this study, we used HTR-8/SVneo cells to construct an in vitro model of ICP to validate the correlation between miR-370-3p and both TM9SF4 and KIT, as well as their association with ICP.

Yinchenhao decoction (YCHD), a traditional Chinese medicine prescription, is widely used to treat various inflammatory diseases, especially liver diseases [20]. It contains three drugs: capillaris (*Artemisia capillaris*), gardenia (*Gardenia jasminoides*), and rhubarb (*Rheum rhabarbarum*) [21]. Previous studies have used high-performance liquid chromatography to identify its effective compounds, including quercetin, scopolamine, chlorogenic acid, and geniposidic acid [21, 22]. Emerging evidence suggests that YCHD may target ICP through multipathway interactions between its bioactive components and the disease's core pathophysiological features. ICP is characterized by disrupted bile acid homeostasis, oxidative stress-induced placental trophoblast apoptosis, and impaired autophagy. Key bioactive compounds in YCHD exhibit targeted effects. Chlorogenic acid in YCHD not only inhibits hepatocyte apoptosis by suppressing mitochondrial oxidative stress but also directly modulates bile acid transporters to alleviate cholestasis [21]. Geniposidic acid, the primary active compound of gardenia, enhances glutathione synthesis and activates Nrf2-mediated antioxidant pathways counteracting the excessive placental reactive oxygen species (ROS) in ICP [23]. Furthermore, quercetin derived from capillaris regulates the PI3K/AKT/mTOR signaling axis [21], a critical pathway implicated in both trophoblast survival and autophagy regulation in ICP. Studies have shown that YCHD can also reduce the expression of TNF, FAS, and Prkcb and regulate the expression of CD14 genes, indicating that it can block the MAPK pathway, which inhibits hepatocyte apoptosis to prevent fibrosis [21]. On the basis of the aforementioned research, we speculate that YCHD can regulate the tricarboxylic acid cycle, increasing oxidative stress levels and related amino acid metabolism, thereby promoting cell apoptosis and autophagy and ultimately producing therapeutic effects on ICP. Studies have also shown that a high dose (12 g/kg) of YCHD has the greatest ameliorative effect on cholestasis, particularly in histopathological examinations, and reduces the levels of alanine transaminase (ALT), aspartate transaminase (AST), and TBA [22]. So, in our experiment, YCHD was orally administered to rats at a dose of 12 g/kg/day (1.2 mL/100 g). However, the precise role of YCHD in ICP has not yet been thoroughly elucidated. This study is aimed at clarifying the effects of YCHD against ICP by establishing an ICP rat model [24], which provides a theoretical and therapeutic basis for the treatment of ICP.

In our study, we aimed to (1) observe the secretion of miR-370-3p associated with ICP utilizing clinical samples and both *in vitro* and *in vivo* ICP models, (2) analyze the relationship between miR-370-3p and its target gene expressions during ICP progression, and (3) determine the possible protection mechanism of YCHD in ICP.

## 2. Materials and Methods

### 2.1. Preparation of YCHD

The YCHD is composed of three herbs: capillaris, gardenia, and rhubarb [22]. The three active ingredients were supplied by Nanjing Pharmaceutical Materials Co., Ltd. (Nanjing, China). The preparation process involves the following steps: capillaris (18 g), gardenia (12 g), and rhubarb (6 g) were weighed and immersed in 360 mL of distilled water (1:10, *w*/*v*) for 12 h. Following this, the mixture was subjected to boiling twice for 1.5 h. The resulting extract was mixed and filtered through filter paper to remove particulates. The filtrate was concentrated to a volume of 36 mL, yielding a mixture containing 1 g of crude drug per milliliter [22]. For *in vivo* assay, the powder was dissolved in water and orally administered to rats at 12 g/kg/day (1.2 mL/100 g) [24].

### 2.2. Clinical Specimen Collection

Clinical serum samples were collected from healthy pregnant women (control) and patients with ICP at the Affiliated Nanjing Hospital of Nanjing University of Chinese Medicine. Written informed consent was obtained from all the participants. The study design was approved by the Ethics Committee of the Affiliated Nanjing Hospital of Nanjing University of Chinese Medicine (Approval No. 2021-LY-ky016). In total nine serum samples from women in late pregnancy were collected: three from normal pregnant women and six from ICP diagnosed women, of which three were mild and three severe cases. The diagnosis of ICP was based on obviously elevated TBAs, mild to moderate elevation of liver function during pregnancy, and cholestasis that returned quickly to normal levels after delivery. According to the “Clinical Diagnosis, Treatment, and Management Guidelines for Intrahepatic Cholestasis of Pregnancy (2024)” by the Obstetrics and Gynecology Branch of the Chinese Medical Association [25], the ICP division is as follows: mild: (1) fasting serum TBA levels in pregnant women range from 10 to 39 *μ*mol/L or postprandial serum TBA levels from 19 to 39 *μ*mol/L and (2) the clinical symptoms are mainly skin itching, with no apparent other symptoms, and severe: (1) pregnant woman's serum TBA levels range from 40 to 99 *μ*mol/L; (2) her serum bilirubin level is higher than the average value; (3) it is accompanied by other conditions, such as multiple pregnancies, preeclampsia, recurrent ICP, or perinatal death caused by ICP; and (4) early-onset ICP. All the clinical serum samples of pregnant women were rapidly frozen, stored in liquid nitrogen, and preserved at − 80°C for further analysis.

### 2.3. In Vitro Cell Culture and Establishment of ICP Model

HTR-8/SVneo cells were obtained from ATCC (number CRL-3271). The cells were cultured in RPMI-1640 medium (Procell) containing 10% fetal bovine serum (FBS) (Procell) and 1% penicillin/streptomycin (Procell). To establish an *in vitro* model of ICP, the cells were subsequently exposed to 100 *μ*mol/L of taurocholic acid (TCA) for 24 h [26], with the experimental design segregated into two groups: HTR-8/SVneo group (control) and HTR-8/SVneo + ICP group. All the cells were propagated at 37°C in an incubator containing 5% carbon dioxide. The cell viability and proliferation rates were assessed via a cell counting kit-8 (CCK-8) (BS350b, Biosharp) according to the manufacturer's instructions.

### 2.4. In Vivo ICP Model and Therapeutic Intervention

Then, 15 pregnant SD rats were purchased from Animal Biotech Industries, housed in a temperature-controlled room with a 12-h light/dark cycle and 60%–65% humidity, and allowed to feed freely. All protocols of animal experiments were conducted in accordance with the Animal Care and Use Committee of the Nanjing University of Chinese Medicine (Approval No. ACU230301). The rats were randomly divided into five groups, with three rats in each group: NC (normal, intraperitoneal injection 2.5 mg/kg physiological saline, days for 5 days); ICP (intraperitoneal injection of estradiol benzoate (EB) 2.5 mg/kg/day for 5 days) [24]; ICP + NC (intraperitoneal injection of EB 2.5 mg/kg/day for 5 days and tail vein injection of 0.1 mg/kg physiological saline for 5 days); ICP + miR-370-3p antagomir (intraperitoneal injection of EB 2.5 mg/kg/day for 5 days + tail vein injection of the miR-370-3p antagomir 0.1 mg/kg for 5 days); and ICP + YCHD (intraperitoneal injection of EB 2.5 mg/kg for 5 days + YCHD orally administered at 12 g/kg/day (1.2 mL/100 g) for 5 days). Serum samples of rats were collected from the tail vein blood before and after injection to measure the levels of ALT, AST, alkaline phosphatase (ALP), and TBA via common biochemical kits (JianCheng Bioengineering Institute). After completing all procedures, pregnant rats were anesthetized via intraperitoneal injection of 3% (*w*/*v*) sodium pentobarbital (30 mg/mL in sterile saline; P3761, Sigma-Aldrich) at 100 mg/kg. Deep anesthesia was confirmed by loss of pedal reflex (no response to toe pinch for > 60 s). Euthanasia was then performed via cervical dislocation under sustained anesthesia, and death was confirmed by the absence of corneal reflex and cessation of heartbeat for > 5 min. This protocol complied with the AVMA Guidelines for Euthanasia of Animals (2020 edition). Immediately after confirming rats death, a midline laparotomy was performed to expose the abdominal cavity. A piece of liver tissue was immediately removed and fixed in 4% paraformaldehyde (PFA) (AS1018, Aspen Biotechnology) for the preparation of paraffin-embedded tissue sections, followed by standard HE staining. The cut placental tissue was thoroughly rinsed with precooled phosphate-buffered saline (PBS) (GNM20012, GENOM), and the sterile dry gauze was used to absorb water. After treatment with RNase-free water, it was quickly placed in liquid nitrogen and preserved at −80°C for further analysis.

### 2.5. Exosome Isolation and Purification

Exosomes in the culture medium (HyClone) or human and rat sera were separated according to the operating instructions in the Total Exosome Isolation Kit (UR52136, Umibio). Initially, an appropriate volume of ExoQuick solution (Umibio) was added to the samples, which were then incubated overnight at 4°C. Subsequently, the samples were centrifuged at 10,000 × *g* for 60 min at 4°C to sediment the exosome-containing fractions. The supernatant was discarded, and the sediment was resuspended in PBS at a ratio of V_PBS : V_initial = 1 : 5. The resuspended solution was transferred to a fresh centrifuge tube and centrifuged again at 12,000 × *g* for 2 min at 4°C. Finally, the supernatant containing the exosome particles was collected and subsequently loaded into the upper chamber of the exosome purification filter (EPF) column. The column was centrifuged at 3000 × *g* for 10 min at 4°C to purify exosomes. The purified exosomes were aliquoted into 50-*μ*L aliquots and stored at −80°C for future use.

### 2.6. Electron Microscopy and Nanoparticle Tracking Analysis (NTA)

The extracted exosomes were dissolved in 2% PFA (P6148, SIGMA) and added 5 *μ*L onto a Formvar carbon-loaded copper mesh (01753-F, PELCO). Subsequently, 100 *μ*L of PBS was dispensed onto the parafilm, and the copper mesh was placed on the PBS droplet using tweezers for rinsing. The mesh was then placed in 1% glutaraldehyde solution (16051, Ted-pella) for 5 min and washed eight times in ddH_2_O_2_ (E130-01A, Novoprotein) for 1 min. The mesh was then exposed to phosphotungstic acid (PTA) (XW120679911, Sinopharm) at a pH of 7 for 5 min and methylcellulose (M0512, SIGMA) on ice for 10 min. Upon completion of these steps, the copper mesh was left to air-dry for 5–10 min before being stored in a box and used for electron microscopy imaging at 80 kV (1230, JEOL).

After dilution with PBS, the extracted exosome samples were introduced into the sample chamber. The NTA (ZetaView PMX 110, Particle Metrix) instrument was calibrated with 110-nm polystyrene microspheres (400168-110, Microtrac) prior to analysis. Subsequently, the diluted exosome samples were subjected to detection using the NTA.

### 2.7. Bioinformatics Analysis

The miRNA components were profiled via high-throughput RNA sequencing analysis (Illumina) in normal pregnant women (control), mild ICP, and severe ICP groups. The samples were sent to Xiamen Life Interconnect Technology Co., Ltd. (Illumina NovaSeq 6000, PE150 sequencing mode). Differentially expressed miRNAs were identified among the three groups. Subsequently, target genes associated with the identified miRNA were screened using the bioinformatics database starBase.

### 2.8. Dual-Luciferase Reporter Assay

When the density of HTR-8/SVneo cells cultured in the medium reaches 80%–90%, plasmid transfection was performed. Specifically, the wild-type (WT) or mutant-type (MUT) fragments of TM9SF4 and KIT were constructed into the pGL6-miR gene plasmid (D2106, ELK Biotechnology), with TM9SF4/KIT 3⁣′UTR (MUT) serving as a control. These constructs, along with miR-370-3p mimics, were transfected into HTR-8/SVneo cells using Lipofectamine 2000 Transfection Reagent (Invitrogen), according to the manufacturer's instructions. Subsequently, the fluorescence intensity of the dual-luciferase reporter assay was measured to validate the targeting relationship between miR-370-3p, KIT, and TM9SF4. The sequences used for plasmid construction are listed in Table S1.

### 2.9. Western Blot Analysis

The exosomes were cleaved using RIPA buffer (AS1004, Aspen Biotechnology) containing a protease inhibitor. An SDS-PAGE kit (AS1012, Aspen Biotechnology) was used for protein separation from purified exosomes extracted from human and rat sera and culture media. After electrophoresis, the proteins on the SDS-PAGE gel were transferred onto a polyvinylidene fluoride (PVDF) membrane (IPVH00010, Millipore), blocked in 5% skimmed milk powder (AS1033, Aspen Biotechnology), and incubated with primary antibodies against CD9 (ab236630, Proteintech), CD63 (25682-1-AP, Proteintech), CD81 (27855-1-AP, Proteintech), TM9SF4 (ab253000, Abcam), KIT (18696-1-AP, Proteintech), or *β*-actin (TDY051, Abcam). After three TBST (TBS with Tween-20) (AS1100, Aspen Biotechnology) washes, the membranes were incubated with secondary antibodies for 30 min. As directed by the manufacturer, protein signals were detected via the ECL method and analyzed via the AlphaEaseFC software processing system.

### 2.10. RNA Extraction and miRNA qRT-PCR Analysis

TRIpure Total RNA Extraction Reagent (EP013, ELK Biotechnology) was used to extract total RNA from the human and rat sera, HTR-8/SVneo cells, and rat placental tissues. EntiLink™ 1^st^ Strand cDNA Synthesis Kit (EQ003, ELK Biotechnology) was used for the synthesis of the first strand cDNA. qRT-PCR was conducted according to the instructions of EnTurbo SYBR Green PCR SuperMix (EQ001, ELK Biotechnology) with the QuantStudio 6 Flex System PCR System (Life Technologies). The fold change was expressed via the 2^−*ΔΔ*Ct^ method. The internal reference for mRNAs was *β*-actin (ELK Biotechnology). The internal parameters of the miRNAs were U6 (ELK Biotechnology). Stem-loop reverse transcription (RT) primers of miRNAs and primers for PCR are listed in Table S1.

### 2.11. miRNA Mimic or Inhibitor Transfection

Transient transfection of miR-370-3p was achieved by transfection with miRNA oligonucleotides (miR-370-3p mimics, inhibitor, and corresponding negative control were synthesized by RiboBio, China). According to the manufacturer's instructions, miR-370-3p mimics, inhibitors, and corresponding negative control were transfected into HTR-8/SVneo cells using the Lipofectamine 2000 Transfection Reagent (Invitrogen). *In vitro* models were divided into HTR-8/SVneo, HTR-8/SVneo+ICP, HTR-8/SVneo+ICP + inhibitor NC, HTR-8/SVneo+ICP + miR-370-3p inhibitor, HTR-8/SVneo+ICP + mimic NC, and HTR-8/SVneo+ICP + miR-370-3p mimics. The miR-370-3p mimics, inhibitor, and corresponding negative control sequences are listed in Table S1.

### 2.12. RNA-Pull Down Assay

Biotinylated miR-370-3p was constructed to generate biotin-miR-370-3p (miR-370-3p probes), and biotinylated miR-NC (NC probes) was used as a control. The cells were divided into HTR-8/SVneo+ICP + NC and HTR-8/SVneo+ICP + miR-370-3p probes, based on the ICP cell models. The cell extracts were incubated with biotinylated RNAs, followed by the addition of agarose beads (Invitrogen) for 1  h. We assess the correlation between miR-370-3p and both TM9SF4 and KIT using an RNA-Binding Protein Immunoprecipitation Kit (Magna RIP). The probe sequences are listed in Table S1.

### 2.13. Statistical Analysis

SPSS software (Version 23.0) was used for the statistical analysis. The results are presented as the means ± SDs from three independent experiments. Differences among groups were evaluated via unpaired Student's *t* test or one-way ANOVA. A *p* value of less than 0.05 indicated a statistically significant difference.

## 3. Results

### 3.1. Enrichment of miR-370-3p in ICP-Secreted Exosomes Is Positively Correlated With Disease Severity

**Figure 1:**
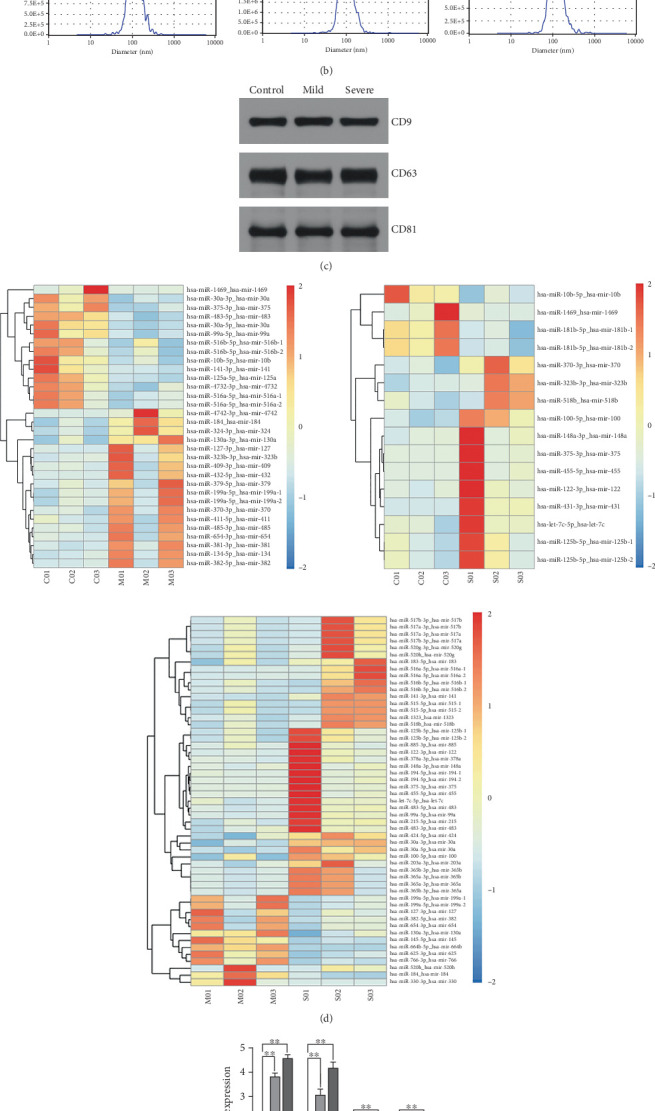
Exosomal miRNAs detected in serum samples from pregnant women. (a) Exosomes secreted by the serum samples of clinical patients were analyzed via electron microscopy. (b) The size of the vesicles secreted by the clinical samples was measured via NTA. (c) Exo-specific markers CD9, CD63, and CD81 were detected via western blotting. (d) Differential expression levels of miRNAs in the exosomes of clinical serum samples from control, mild ICP, and severe ICP patients. (e) Relative expression of miR-370-3p, miR-323b-3p, miR-1469, and miR-10b5p, in the three groups. ⁣^∗^*p* < 0.05 and ⁣^∗∗^*p* < 0.01 vs. the control group.

In our initial experiment, we successfully isolated exosomes from serum samples of individuals in the control, mild, and severe ICP groups. We subsequently analyzed the exosomes via electron microscopy and NTA. Our observations revealed a distinct presence of highly condensed particles within the exosomes, displaying diameters spanning from 30 to 100 nm (Figure 1a,b). These findings confirm that patients with ICP secrete exosomes into their serum. Furthermore, we assessed the abundance of secretory protein markers, including CD9, CD63, and CD81, using western blot analysis. Interestingly, our analysis revealed no notable variations in the levels of these markers among the different ICP severity groups (Figure 1c).

Moreover, we employed a bioinformatic tool to search for miRNAs that are pivotal to serum secretion. Our analysis identified multiple miRNAs within serum-derived exosomes from patients with ICP (Figure 1d). Subsequently, qRT-PCR analysis revealed three clinical comparison groups of differentially expressed miRNAs: miR-370-3p, miR-323b-3p, miR-1469, and miR-10b-5p. Notably, miR-370-3p emerged as the most significantly differentially expressed miRNA among these, prompting its selection as the primary target miRNA for subsequent in-depth investigation (Figure 1e).

### 3.2. TM9SF4 and KIT Directly Interact With miR-370-3p

**Figure 2:**
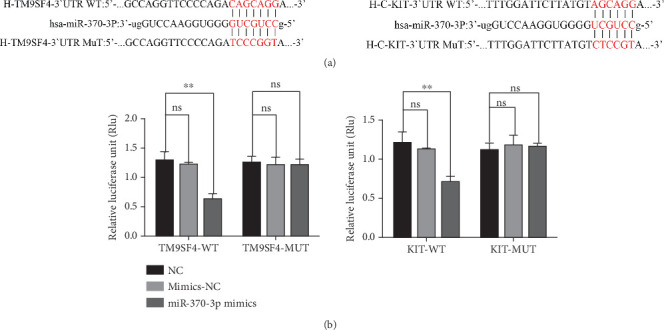
The relationship of TM9SF4 and KIT with miR-370-3p. (a) Sequence alignment of miR-370-3p with the binding sites within the WT or MUT regions of TM9SF4 and KIT. (b) The relative luciferase activity of the 3⁣′UTR TM9SF4/KIT-WT or 3⁣′UTR TM9SF4/KIT-MUT with miR-370-3p mimics. ⁣^∗^*p* < 0.05 and ⁣^∗∗^*p* < 0.01 vs. the NC group. ns means not significant.

Using the bioinformatics tool starBase, we predicted the potential binding sites of miR-370-3p and identified TM9SF4 and KIT as candidate targets for further investigation. To validate these predictions, we used a dual-luciferase reporter assay. Our results demonstrated that the miR-370-3p mimics significantly reduced luciferase activity in constructs containing the wild-type 3⁣′UTR of both TM9SF4 and KIT (3⁣′UTR TM9SF4-WT and 3⁣′UTR KIT-WT) compared to the mimics NC and NC groups (Figure 2). Conversely, no notable change in luciferase activity was observed in constructs harboring the mutated 3⁣′UTR of TM9SF4 and KIT (3⁣′UTR TM9SF4-MUT and 3⁣′UTR KIT-MUT), confirming that miR-370-3p directly targets TM9SF4 and KIT.

### 3.3. miR-370-3p Overexpression and Low TM9SF4/KIT Expression in ICP-Secreting HTR-8/SVneo Cells

**Figure 3:**
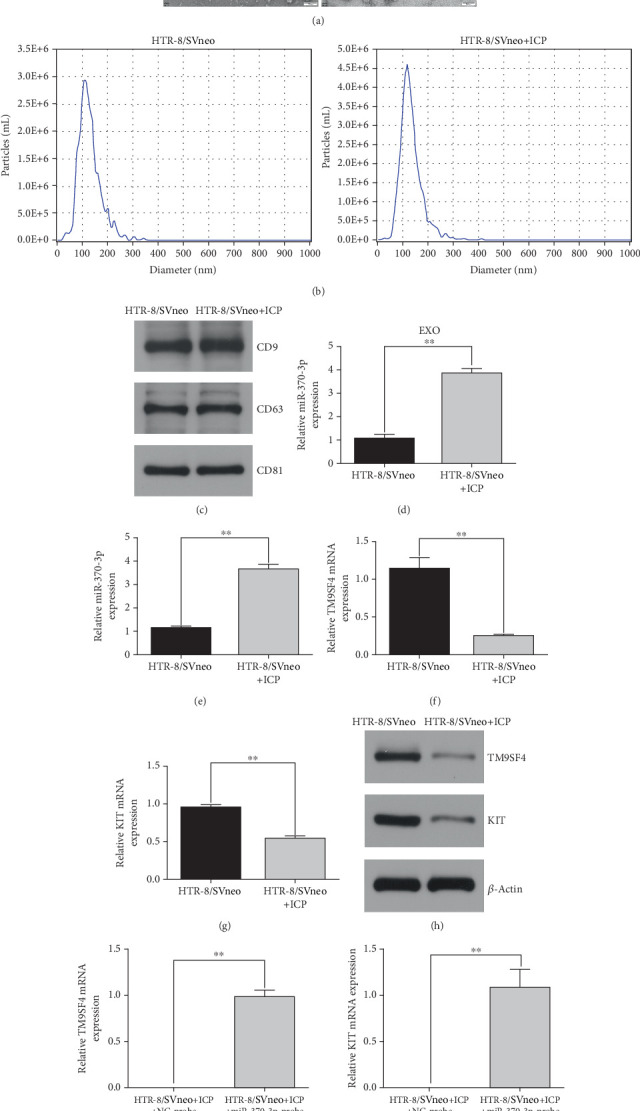
Expressions of miR-370-3p, TM9SF4, and KIT in ICP-secreting HTR-8/SVneo cells. (a) Exosomes secreted by HTR-8/SVneo cells were identified via electron microscopy. (b) NTA was used to measure the size of the vesicles. (c) Western blot analysis of exo-specific proteins. (d) The expression of miR-370-3p in the supernatant exosomes of the HTR-8/SVneo cell and HTR-8/SVneo+ICP cell via qRT-PCR analysis. (e–g) Levels of miR-370-3p, TM9SF4, and KIT were detected via qRT-PCR analysis in the HTR-8/SVneo cell and HTR-8/SVneo+ICP cell groups. (h) TM9SF4 and KIT expressions in the HTR-8/SVneo and HTR-8/SVneo+ICP groups were detected via western blotting. (i) The relationships between miR-370-3p and TM9SF4 or KIT were verified via an RNA pull-down assay in the HTR-8/SVneo+ICP + NC probe group and the HTR-8/SVneo+ICP + miR-370-3p probe group. ⁣^∗^*p* < 0.05 and ⁣^∗∗^*p* < 0.01 vs. the control group.

We isolated exosomes from both normal and ICP-secreting HTR-8/SVneo cells (Figures 3a, 3b, and 3c) and subsequently analyzed the expression of miR-370-3p in these cells using qRT-PCR. Our results showed that ICP-secreting HTR-8/SVneo cells exhibited significantly higher levels of miR-370-3p compared to normal HTR-8/SVneo cells (Figure 3d,e). Furthermore, qRT-PCR and western blot assays revealed that ICP-secreting cells had reduced the expressions of TM9SF4 and KIT compared to normal cells (Figures 3f, 3g, and 3h). To validate the direct interaction between miR-370-3p and its targets, TM9SF4 and KIT, we performed an RNA pull-down assay. This assay confirmed that cells transfected with miR-370-3p probes significantly upregulated TM9SF4 and KIT expressions compared to cells transfected with NC probes (Figure 3i). These findings suggest that miR-370-3p plays a role in ICP by regulating TM9SF4 and KIT expressions.

### 3.4. miR-370-3p Mediated Downregulation of TM9SF4 and KIT in ICP Cell Models

**Figure 4:**
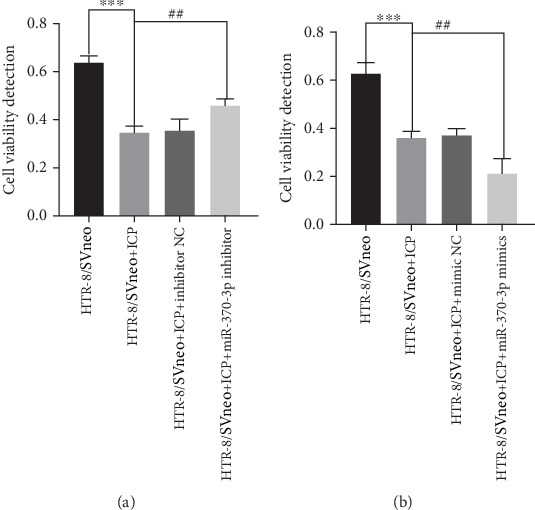
(a, b) Cell viability detection. ⁣^∗∗∗^*p* < 0.001 vs. the control group. ^##^*p* < 0.01 vs. the control group.

Quantitative analysis of cell viability and proliferation dynamics revealed marked suppression of cellular growth in ICP-modeled HTR-8/SVneo cells, whereas cells treated with miR-370-3p inhibitors exhibited a lesser degree of reductions (Figure 4a). Notably, transfection with miR-370-3p mimics induced the most significant attenuation of proliferative capacity compared to other experimental groups (Figure 4b).

**Figure 5:**
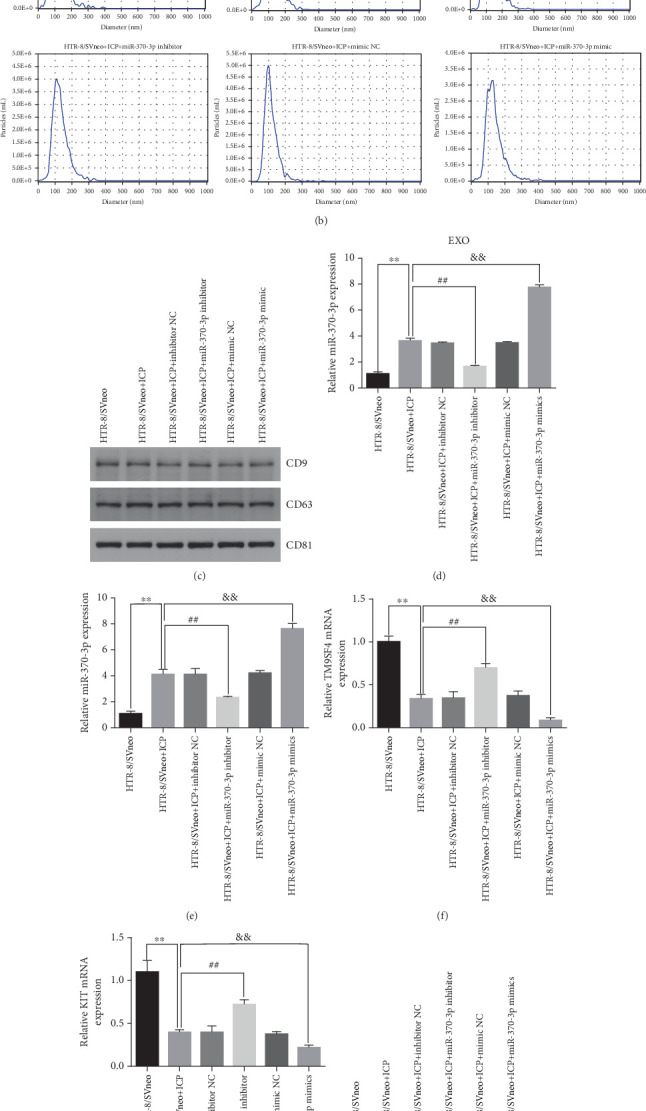
Expression of miR-370-3p, TM9SF4, and KIT in six groups of HTR-8/SVneo cells. (a) Exosomes secreted by HTR-8/SVneo cells were identified via electron microscopy. (b) NTA measured the size of the vesicles. (c) Western blot analysis of exo-specific proteins. (d–g) The levels of miR-370-3p, TM9SF4, and KIT were assayed by qRT-PCR. (h) Evaluation of TM9SF4 and KIT expressions via western blotting. ⁣^∗^*p* < 0.05 and ⁣^∗∗^*p* < 0.01 vs. the control group. ^#^*p* < 0.05 and ^##^*p* < 0.01 vs. the control group. ^&^*p* < 0.05 and ^&&^*p* < 0.01 vs. the control group.

We successfully isolated and characterized exosomes from various sets of HTR-8/SVneo cells (Figures 5a, 5b, and 5c). We then evaluated the expression levels of miR-370-3p, TM9SF4, and KIT in six groups of these cells using qRT-PCR and western blotting. miR-370-3p was overexpressed in ICP-secreting HTR-8/SVneo cells, with maximum expression observed with miR-370-3p mimics. In contrast, HTR-8/SVneo cells transfected with an miR-370-3p inhibitor exhibited a decrease in miR-370-3p levels compared to those in the inhibitor NC group (Figure 5d,e). Further validation revealed that the inhibition of miR-370-3p led to a significant increase in both TM9SF4 and KIT expressions in ICP-secreting HTR-8/SVneo cells compared to the inhibitor NC group (Figures 5f, 5g, and 5h). These findings confirmed that miR-370-3p exerts a negative regulatory effect on the expressions of TM9SF4 and KIT in ICP-secreting HTR-8/SVneo cells.

### 3.5. YCHD: Modulating miR-370-3p via the TM9SF4/KIT Pathway to Protect Against ICP

**Figure 6:**
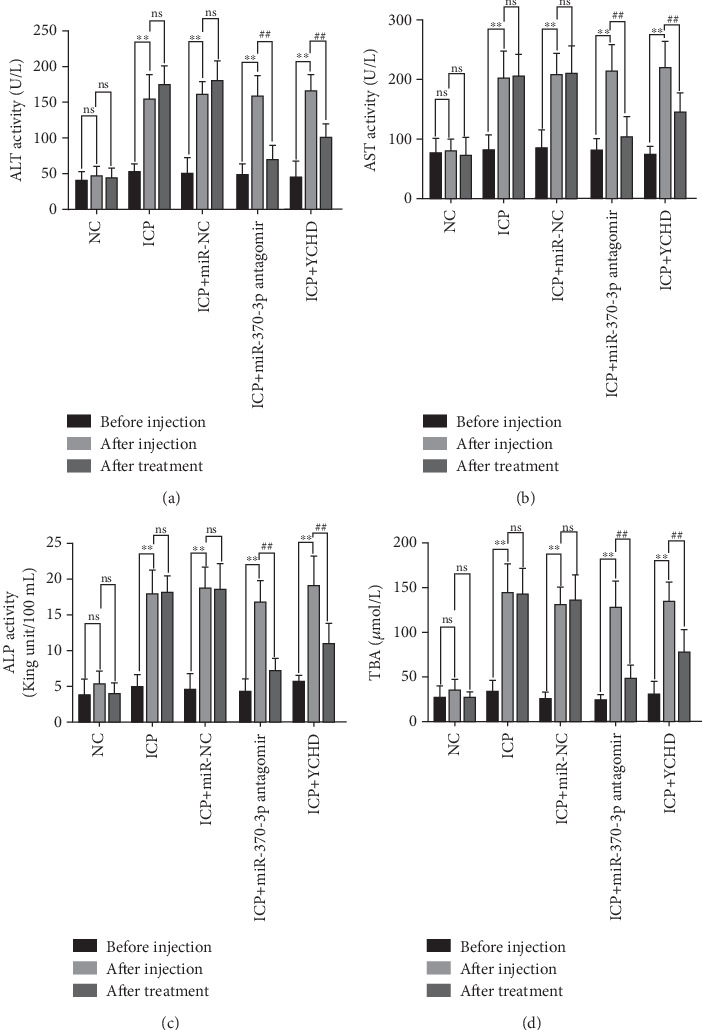
(a–d) ALT, AST, ALP, and TBA levels in five groups of rats. ⁣^∗∗^*p* < 0.01 vs. after injection group. ^##^*p* < 0.01 vs. after injection group. ns means not significant.

An ICP animal model was established through in vivo administration of 2.5 mg/kg EB daily for 5 consecutive days [24], which led to significant elevations in the rat serum levels of ALT, AST, ALP, and TBA. To investigate potential therapeutic interventions, we employed miR-370-3p antagomir, a chemically modified RNA oligonucleotide designed to specifically inhibit the function of miR-370-3p. Our results demonstrate that treatment with both YCHD and miR-370-3p antagomir resulted in a significant improvement of these elevated biochemical markers, suggesting their potential therapeutic effects against ICP-induced liver injury (Figures 6a, 6b, 6c, and 6d). This finding underscores the importance of miR-370-3p in the pathogenesis of ICP-related liver injury.

Before injection: This denotes the baseline conditions of five groups of rats prior to any treatment intervention. After injection: The NC group received intraperitoneal injections of 2.5 mg/kg physiological saline for 5 days, while the ICP groups underwent intraperitoneal injections of 2.5 mg/kg EB daily for 5 days to establish ICP models. This serves as the reference point for comparing subsequent treatment effects. After treatment: This denotes the final outcome after completion of the entire treatment protocol in five groups of rats.

**Figure 7:**
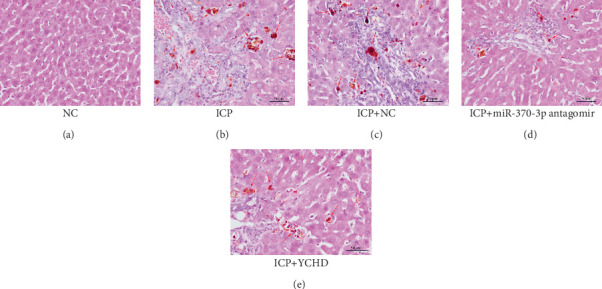
(a–e) Pathological liver sections from the five groups of rats.

The observation of liver slices from pregnant rats via light microscopy revealed no significant morphological changes in the livers of the NC group, and the hepatic lobule structure was clear. The arrangement of liver cells in the ICP group was slightly disordered, with some liver cells exhibiting vacuolar and granular degeneration and a small amount of necrosis (Figure 7a,b). To substantiate the correlation between miR-370-3p and YCHD in ICP, the liver slice results further revealed that the ICP + miR-370-3p antagomir and ICP + YCHD groups presented fewer liver cell lesions than did the ICP + NC group, indicating the therapeutic potential of YCHD in ICP management (Figure 7c, 7d, and 7e).

**Figure 8:**
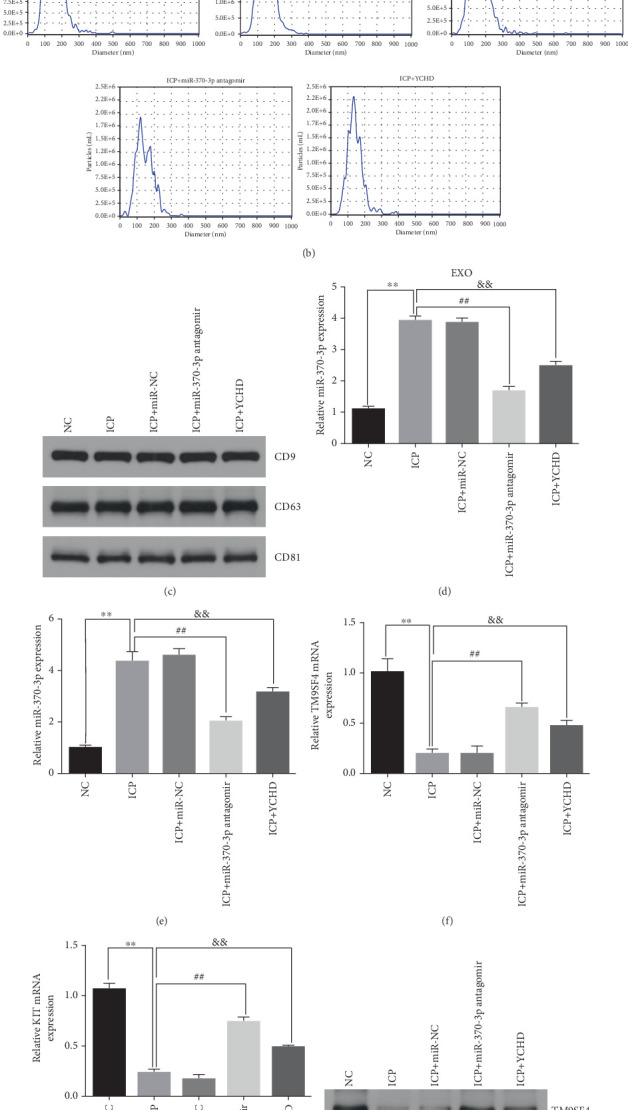
Yinchenhao decoction regulates miR-370-3p, TM9SF4, and KIT levels in ICP. (a) Exosomes secreted by rat serum were identified via electron microscopy. (b) NTA was used to measure the vesicle size. (c) Western blot analysis of exo-specific proteins. (d) The levels of miR-370-3p in serum exosomes were determined via qRT-PCR analysis. (e–g) The levels of miR-370-3p, TM9SF4, and KIT in placental tissues were determined via qRT-PCR analysis. (h) The expressions of TM9SF4 and KIT in placental tissues were determined via western blotting. ⁣^∗^*p* < 0.05 and ⁣^∗∗^*p* < 0.01 vs. the control group. ^#^*p* < 0.05 and ^##^*p* < 0.01 vs. model group. ^&^*p* < 0.05 and ^&&^*p* < 0.01 vs. the model group.

Similarly, exosomes were collected from rat serum and identified via a previously described method (Figures 8a, 8b, and 8c). We measured the levels of miR-370-3p, TM9SF4, and KIT in the serum and placental tissues of ICP rats. Our data revealed that miR-370-3p expression increased in the ICP model group, whereas KIT and TM9SF4 mRNA levels decreased. The YCHD and miR-370-3p antagomir groups reversed these trends, with decreased miR-370-3p expression and restored TM9SF4 and KIT levels (Figure 8d, 8e, 8f, 8g, and 8h). Thus, miR-370-3p exerts a pivotal regulatory influence on TM9SF4 and KIT, as demonstrated by genetic and proteomic analyses. YCHD regulates miR-370-3p during ICP progression by targeting the TM9SF4/KIT pathway, thereby conferring protection against ICP via the miR-370-3p/TM9SF4/KIT axis.

## 4. Discussion

ICP is a prevalent pregnancy-associated liver disease linked to fetal distress, premature delivery, and the frequency of sudden intrauterine fetal death [27]. TBA has poor specificity and sensitivity; however, it is currently the primary diagnostic and screening indicator in the clinic. Among the numerous noncoding RNAs that regulate cell proliferation, differentiation, apoptosis, and other aspects of the cell life cycle, miRNA has been a hot research topic in recent years. Therefore, our study is aimed at screening and identifying miRNAs associated with ICP through the use of clinical samples and the construction of ICP in in vivo and in vitro models, as well as analyzing the role of target miRNA and their target genes in the development of ICP. These findings provide a basis for the clinical treatment of ICP.

Exosomes are extracellular vesicles that contain miRNAs. The proteins they carry are involved in the physiological and pathological processes of many diseases that promote degradation of target mRNAs or inhibit their translation [28, 29]. Initially, we used differential centrifugation to isolate exosomes from clinical serum samples of pregnant women. We then identified these exosomes via electron microscopy, NTA, and western blotting. Second, we sequenced the exosomal miRNAs in the serum of pregnant women from normal (control), mild, and severe ICP groups to determine which miRNAs are responsible for the ICP phenotype. Our data revealed several differentially expressed miRNAs across the three groups, including miR-370-3p, miR-323b-3p, miR-1469, and miR-10b5p. Among these four miRNAs, miR-370-3p presented the most significantly altered expression according to qRT-PCR analysis. Therefore, miR-370-3p was identified as an interesting candidate gene.

Next, we investigated the latent targets of miR-370-3p in ICP. On the basis of starBase and dual-luciferase reporter assays, we found direct interactions between miR-370-3p and both TM9SF4 and KIT. Additionally, RNA pull-down experiments corroborated that TM9SF4 and KIT are indeed target genes of miR-370-3p. We collected exosomes from the ICP-secreted HTR-8/SVneo cells and normal HTR-8/SVneo cells to verify the expressions of miR-370-3p, TM9SF4, and KIT via qRT-PCR or western blotting. The results demonstrated that miR-370-3p is involved in ICP by targeting TM9SF4 and KIT. Subsequently, we transfected HTR-8/SVneo cells with miR-370-3p inhibitors or miR-370-3p mimics and further verified the targeting regulatory effects of miR-370-3p on TM9SF4 and KIT via in vitro cellular experiments.

In recent years, the availability of interventional ICP medicines has been limited. With the popularization of Chinese medicine and more comprehensive clinical applications, the pharmacological effects of YCHD on liver protection, choleresis, the regulation of blood lipids, and immunity have been recognized [30]. In spite of the paucity of knowledge pertaining to the healing mechanism of YCHD in ICP, this study sought to examine whether YCHD holds protective properties against this ailment. This investigation involved the creation of a rat model of ICP, wherein the elevation of liver function markers and the alterations in the liver's pathological state served as reliable indicators of successful induction. Similarly, exosomes were successfully collected and identified from rat serum via previously described methods. In addition, we observed that YCHD reversed the changes in the expressions of miR-370-3p, TM9SF4, and KIT in the serum exosomes and placental tissues of ICP-induced rats. These findings indicate that YCHD regulates miR-370-3p levels during ICP progression by targeting the TM9SF4 and KIT genes.

In conclusion, our investigation revealed the protective function of YCHD against ICP through regulation of the miR-370-3p/TM9SF4/KIT axis. Our findings provide a robust empirical basis for further exploration of the therapeutic potential of YCHD in ICP treatment.

### 4.1. Limitations

While this study provides valuable insights into the therapeutic potential of YCHD in ICP, several limitations should be acknowledged. First, as a multicomponent herbal formulation, YCHD's pharmacological effects may extend beyond the identified miR-370-3p/TM9SF4/KIT-mTOR axis due to potential synergistic or antagonistic interactions among its bioactive compounds (e.g., quercetin, chlorogenic acid, and geniposidic acid). Future network pharmacology and systems biology approaches will be essential to comprehensively map YCHD's polypharmacology and identify potential off-target effects. Second, the experimental findings were derived from a rodent ICP model, which may not fully recapitulate the heterogeneity of human ICP pathophysiology. Future clinical studies involving larger patient cohorts are required to validate the translational relevance of our results, particularly regarding dose optimization and interindividual variability in treatment response. Third, while the selected YCHD dose (12 g/kg) was based on prior pharmacodynamic evidence, potential long-term safety concerns and tissue-specific effects remain to be explored. Addressing these limitations will refine our understanding of YCHD's role in ICP management and guide its clinical application.

### 4.2. Future Perspectives and Challenges

These findings lay a foundation for translating YCHD's therapeutic potential into clinical practice for ICP management. Future research should prioritize the following steps: First, dose optimization studies in nonhuman primates or human organoids are warranted to bridge the gap between rodent models and clinical applications, particularly given the complexity of YCHD's multicomponent nature. Second, multicenter clinical trials should evaluate YCHD's efficacy and safety in ICP patients, with stratification based on disease severity and biomarker profiles. Third, standardization of YCHD preparation is critical to ensure batch-to-batch consistency in bioactive compound ratios, which could be achieved through advanced quality control protocols integrating metabolomics and HPLC fingerprinting.

## Data Availability

The data that support the findings of this study are available from the corresponding author upon reasonable request.

## Nomenclature

ICP: intrahepatic cholestasis of pregnancy
NTA: nanoparticle tracking analysis
qRT-PCR: quantitative reverse transcription PCR
ALT: alanine transaminase
AST: aspartate transaminase
ALP: alkaline phosphatase
TBA: total bile acid
TCA: taurocholic acid
FBS: fetal bovine serum
PTA: phosphotungstic acid
PBS: phosphate-buffered saline
PFA: paraformaldehyde
EB: estradiol benzoate
WT: wild type
MUT: mutant type

## Supporting Information

Supporting Information Additional supporting information can be found online in the Supporting Information section. Table S1: This table includes the primers for the construction of luciferase reporter plasmid, the stem-loop reverse transcription (RT) primers of miRNAs, and primers for PCR, the sequences of miRNA mimics, inhibitor, and corresponding negative control and the sequences of probes.
