# Supplementary material for: Yinchenhao Decoction Protects Against Intrahepatic Cholestasis During Pregnancy Through the miR-370-3p/TM9SF4/KIT Axis
Source: Biomed Res Int. 2025 Jul 26;2025:3000226. doi: 10.1155/bmri/3000226 (PMC12317817; doi:10.1155/bmri/3000226)
Supplement: Supporting Information — Additional supporting information can be found online in the Supporting Information section. Table S1: This table includes the primers for the construction of luciferase reporter plasmid, the stem-loop reverse transcription (RT) primers of miRNAs, and primers for PCR, the sequences of miRNA mimics, inhibitor, and corresponding negative control and the sequences of probes. [file 3000226.f1.docx]

**Supplemental Table 1**

This table includes the primers for construction of luciferase reporter plasmid, the stem-loop reverse transcription (RT) primers of miRNAs and primers for PCR, the sequences of miRNA mimics, inhibitor and corresponding negative control, and the sequences of probes.

| **Primers for construction of luciferase reporter plasmid** | |
| --- | --- |
| TM9SF4-wild type | F: GCCAGGTTCCCCAGACAGCAGGA |
|  | R: AGGCTGCTGTCTGGGGAACCTGGCC |
| TM9SF4-mutant type | F: GCCAGGTTCCCCAGATCCCGGTA |
|  | R: TACCGGGATCTGGGGAACCTGGCCG |
| KIT-wild type | F: TTTGGATTCTTATGTAGCAGGA |
|  | R: AGGCTAGCATAAGAATCCAAAA |
| KIT-mutant type | F: TTTGGATTCTTATGTCTCCGTA |
|  | R: TACGGAGACATAAGAATCCAAAAT |

| **Primers for PCR** | |
| --- | --- |
| miR-370-3p | RT: CTCAACTGGTGTCGTGGAGTCGGCAATTCAGTTGAGACCAGGTT |
|  | F: AATTGCCTGCTGGGGTGG |
|  | R: CTCAACTGGTGTCGTGGAGTC |
| miR-323b-3p | RT:CTCAACTGGTGTCGTGGAGTCGGCAATTCAGTTGAGAAGAGGTC |
|  | F: CCGCCCAATACACGGTCG |
|  | R: CTCAACTGGTGTCGTGGAGTC |
| miR-1469 | RT: CTCAACTGGTGTCGTGGAGTCGGCAATTCAGTTGAGGGAGCCCG |
|  | F: AATTTCTCGGCGCGGGG |
|  | R: CTCAACTGGTGTCGTGGAGTC |
| miR-10b-5p | RT: CTCAACTGGTGTCGTGGAGTCGGCAATTCAGTTGAGCACAAATT |
|  | F: GGCCTACCCTGTAGAACCG |
|  | R: CTCAACTGGTGTCGTGGAGTC |
| TM9SF4 | F: ACCAGGAGCAAACCCAAGTC |
|  | R: CTTGGCACAAAAACAGTTCACT |
| KIT | F: ATGGCATTGTACTCAATGGATTT |
|  | R: CCTCCAAATATTTGTCTACACACAT |
| U6 | F: CTCGCTTCGGCAGCACAT |
|  | R: AACGCTTCACGAATTTGCGT |
| β-actin | F: GTCCACCGCAAATGCTTCTA |
|  | R: TGCTGTCACCTTCACCGTTC |

| **miRNA mimics, inhibitor and negative control mimics** | |
| --- | --- |
| negative control mimics | F: UUUGUACUACACAAAAGUACUG |
|  | R: CAGUACUUUUGUGUAGUACAAA |
| negative control inhibitor | F: GACUAAAUUACACUACUGUAAA |
|  | R: AAUGUAGUGUAAUUUAGUCG |
| miR-370-3p mimics | F: GCCUGCUGGGGUGGAACCUGGU |
|  | R: ACCAGGUUCCACCCCAGCAGGC |
| miR-370-3p inhibitor | F: ACCAGGUUCCACCCCAGCAGGC |
|  | R: GCCUGCUGGGGUGGAACCUGGU |

| **Probes** | |
| --- | --- |
| miR-370-3p probe | GCCUGCUGGGGUGGAA |
| NC probe | UUUGUACUACACAAAA |

**Abbreviations:** RT, Reverse transcription F, Forward R, Reverse.
